# Optical coherence tomography angiography in patients with diabetic retinopathy treated with anti-VEGF intravitreal injections: Case report

**DOI:** 10.1097/MD.0000000000008379

**Published:** 2017-11-10

**Authors:** Katarzyna Michalska-Małecka, Anna Heinke Knudsen

**Affiliations:** a Department of Ophthalmology, School of Medicine in Katowice; b Professor K. Gibinski University Clinical Center, Medical University of Silesia, Katowice, Poland.

**Keywords:** anti-VEGF, diabetic macular edema, diabetic retinopathy, optical coherence tomography angiography

## Abstract

**Purpose:**

To present optical coherence tomography angiography (OCTA) features in patients with diabetic retinopathy (DR) at the baseline and in response to treatment with anti-VEGF intravitreal injections. To investigate the role of OCTA in management of patients with DR.

**Methods:**

Retrospective case series showing primary outcomes of 3 patients with DR and diabetic macular edema. Patients were injected intravitreally a loading phase of 3 monthly 2.0 mg aflibercept, followed by 2 injections bimonthly (5 injections in total). Before each injection OCTA was performed using 3 mm × 3 mm scans (Optovue, XR Avanti). The obtained scans of the macula were analyzed and compared to the image at the baseline. Best-corrected visual acuity (BCVA) was examined at the baseline and before each injection.

**Results:**

In the superficial plexus, a rarefaction of capillaries with capillary dropout and nonperfusion areas were present in all eyes. The microaneurysms were good to visualize in 3 mm × 3 mm scans. In deep vascular network, evident microvascular alterations around the small cystoid edema cells were to detect. There were no differences in perfusion density level for the whole macular area in 3 mm × 3 mm scans shown in density maps between injections in all presented cases. After a series of aflibercept intravitreal injections decreased cystic changes were observed. Moreover in all presented cases, the decrease in central retinal thickness that correlated clinically with improvement of visual acuity (BCVA) was observed. All patients achieved a goal of well-controlled diabetes by having a HbA1c level (<8.0%) before each injection.

**Conclusions:**

OCTA is a dyeless, quick, and noninvasive method which allows to detect ischemic changes in DR and might be a useful tool in observing the progress of the disease and the response to anti-VEGF treatment in clinical practice.

## 1 Introduction

Diabetic retinopathy (DR) is the leading cause of visual impairment and blindness in adults in working age worldwide.^[1,2]^ Since the number of patients suffering from DR is expected to grow, further investigations are needed regarding early detection of microvascular changes and new diagnostic methods allowing evaluation of treatment effects.

DR is microangiopathy characterized by a pericyte loss, formation of microaneurysms, and a breakdown of the blood–retinal barrier with vascular hyperpermeability.^[3]^ Vascular endothelial growth factor (VEGF) is a significant factor in the development of proliferative diabetic retinopathy (PDR) and diabetic macular edema (DME).^[4,5]^ VEGF is altering retinal capillary permeability by increasing the phosphorylation of proteins involved with tight junctions.^[5,6]^ VEGF production is induced in response to hypoxia or ischemia and it is considered as the primary factor involved in neovascularization in PDR.^[5,7]^ The interest of using anti-VEGF in cases of DME as a primary treatment is recently increasing, since the laser therapy, which still remains the gold standard for PDR and DME treatment, is causing more extensive tissue damage.^[5]^

The dilated slit lamp fundus examination is the gold standard to screen for DR. Although fluorescein angiography (FA) is more sensitive than biomicroscope examination to detect early microvascular changes, it is an invasive procedure that requires the administration of intravenous dye which can lead to some adverse effects. Therefore, this technique is not optimal for screening or performing frequent longitudinal assessments.^[8–10]^ Studies of the foveal microvasculature using FA have shown enlargement of intercapillary areas in DR,^[11]^ but the ability to detect microvasculature alterations has been limited by the leakage and superposition of the capillary networks.^[12]^ Optical coherence tomography angiography (OCTA) is a new modality that safely, quickly, and noninvasively shows the retinal microvasculature with resolution that exceeds FA.^[12–17]^ OCTA allows separating the superficial and deep vascular plexus, whereas the deep capillary network is barely visible on FA.^[14,17]^ The use of this new technology to study microvascular changes in DR seems therefore meaningful. OCTA can visualize impaired capillary perfusion, neovascularization, intraretinal vascular anomalies, some types of microaneurysms and intraretinal fluid with equal or better resolution than conventional FA.^[18–22]^ OCTA allows also to provide a 3-dimensional mapping of the retina and choroidal microvasculature and vascular mapping of the macular perfusion.^[17,23,24]^

The aim of this study was to assess foveal microvascular changes in patients with DR at the baseline and in response to treatment with anti-VEGF intravitreal injections using OCTA as well as to investigate the role of this novel imaging method in management of patients with DR.

## 2 Methods

The presented retrospective case series include 3 patients with DR treated in Professor K. Gibinski University Clinical Center, Medical University of Silesia, Katowice, Poland, between May 2016 and January 2017. Informed consent was obtained routinely from all examined patients to participate in this research. This study was performed in compliance with Declaration of Helsinki. The research was approved by Bioethics Committee of the Medical University of Silesia in Katowice (KNW/0022/KB1/38/III/15/16).

Inclusion criteria embraced: patient's age above 18 years old, presence of diabetes mellitus (DM) (type I or type II), diagnosis of DR with a visual acuity deterioration due to DME. Data collected included baseline demographics (gender, mean duration of diabetes, hemoglobin A1c, and medical history) and current ophthalmologic examination findings (best-corrected visual acuity (BCVA), slit-lamp biomicroscopy, fundus examination, FA, and OCTA). Patients with BCVA of 0.6-0.1 Snellen charts (≥30 letters or ≤78 letters in Early Treatment Diabetic Retinopathy Study (ETDRS)) were included in the study. Central retinal thickness (CRT) was assessed in OCTA and had to be in an interval between 325 and 600 μm. If DME was present in both eyes, the eye with higher CRT was included in the study. In presented case series all 3 patients met the inclusion criteria only for 1 eye.

Patients with any other retinal disorders, presence of media opacities such as vitreous hemorrhage, as well as endophthalmitis, uveitis, uncontrolled glaucoma, intraocular pressure (IOP) >24 mm Hg, vitreoretinal tractions, active untreated PDR, panretinal photocoagulation (PRP) or grid laser within the last 3 months, intravitreal injections of anti-VEGF or steroids within the last 3 months, any history of vitrectomy in treated eye, comorbidities such as: age-related macular degeneration (AMD), high degenerative myopia, retinal vascular occlusions, retinal detachment, choroid neovascularization, iris neovascularization and those with general contraindications for anti-VEGF intravitreal injections, were excluded from the study.

Patients with DR and DME were injected intravitreally a loading phase of 3 monthly 2.0 mg aflibercept, followed by 2 injections bimonthly (5 injections in total). Before each injection OCTA was performed using 3 mm × 3 mm scans (Optovue, XR Avanti). The obtained scans of the macula were analyzed and compared to the image at the baseline. BCVA was examined at the baseline and before each injection.

**Figure 1:**
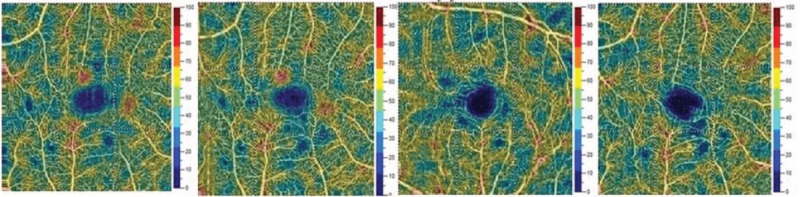
The picture is showing vascular density color perfusion maps generated for superficial capillary layer. In the color maps, bright red represents a density of greater than 50% perfused vessels, dark blue represents no perfused vessels, and intermediate perfusion densities are color coded accordingly.

The AngioVue OCTA device (Optovue, Inc., Freemont, CA) was used to obtain amplitude decorrelation angiography images. The scanning area was captured in 3 mm × 3 mm sections centered on the fovea. Present parameters were used to segment the capillary bed in the superficial and deep capillary plexus (DCP). The en face images of the superficial capillary plexus (SCP) were segmented with an inner boundary at 3 mm beneath the inner limiting membrane and an outer boundary at 15 mm beneath the inner plexiform layer. The en face images of the DCP were segmented with inner and outer boundaries at 15 and 70 mm, respectively, beneath the inner plexiform layer. Qualitative data consisted of retinal vascular density color perfusion maps generated for each microvascular layer. In the color maps (Fig. 1), bright red represents a density of greater than 50% perfused vessels, dark blue represents no perfused vessels, and intermediate perfusion densities are color coded accordingly.

The HbA1c level was assessed before each injection. The level below 8% was considered as the goal in optimal management of diabetes and DR according to Polish Diabetes Association.^[25]^

## 3 Results

**Figure 2:**
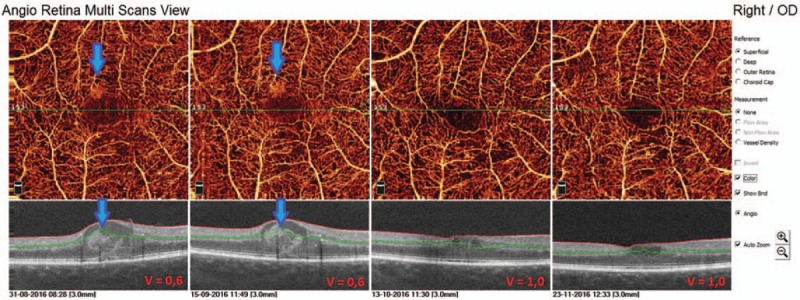
Multiscan view of OCTA in 52-year-old man with type 1 diabetes and nonproliferative diabetic retinopathy (NPDR) at the baseline and after each injection. The arrows are showing exudates in superficial capillary plexus in angioflow and OCT scans.

**Figure 3:**
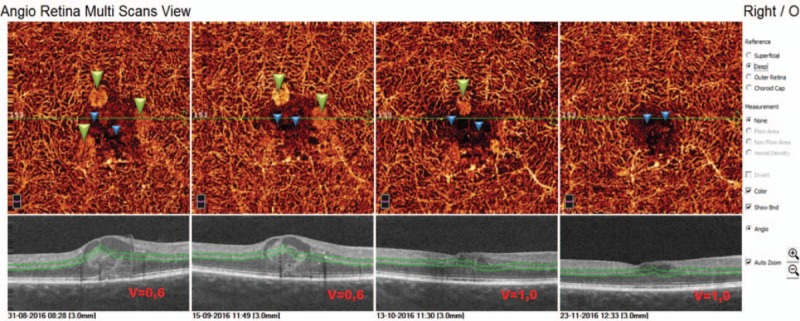
Multiscan view of OCTA showing deep capillary plexus in 52-year-old patients with type 1 diabetes before and after intravitreal aflibercept injections. The regular architecture of capillary vortexes is altered by exudates (green triangles) and cysts of edema (blue triangles). After third injection decrease in cystic changes is to observe in angioflow images.

In the first presented case (52-year-old male with DR and DME) we observe microvascular changes in OCTA scans of superficial and DCP. The patient was diagnosed with DM type 1 and treated with insulin for 20 years. The patient underwent 3 monthly anti-VEGF injections. The abnormal superficial plexus with rarefaction of capillaries and nonperfusion areas outside foveal avascular zone (FAZ) are visible (Fig. 2). In both angioflow images and B-scan OCT the exudates accompanied by retinal edema are to observe. The multiscan view of DCP shows that the normal architecture and regular pattern of the capillary vortexes was altered by the hyporeflective cysts of edema and hyperreflective foci of exudates (Fig. 3). After third injection, some normalization in the capillaries structure and decrease in cystic changes in DCP were observed, the exudates were no more visible and CRT has decreased substantially from 462 μm at the baseline to 298 μm after 3rd aflibercept injection. This result correlated with clinical visual outcome. The BCVA improved from 0.6 to 1.0 already after second anti-VEGF injection. The HbA1c level in this patient was between 7.3% and 7.8%. The summary of vessel density, CRT, and HbA1c values are presented in Table 1. The whole image vessel density in presented case was at the mean value of 51, 62% perfused vessels. The table is showing the gradual decrease in CRT after each injection. HbA1c levels before each injection are between 7.3% and 7.8%. In vessel perfusion maps for the whole macular image there were no difference in vessel density after each injection (Fig. 1).

**Table 1:**
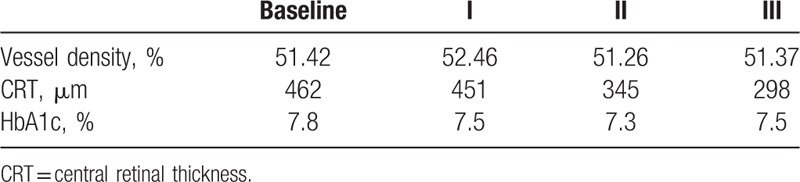
The summary of vessel density, CRT, and HbA1c values at baseline, after 1st, 2nd, and 3rd injection in first presented case.

**Figure 4:**
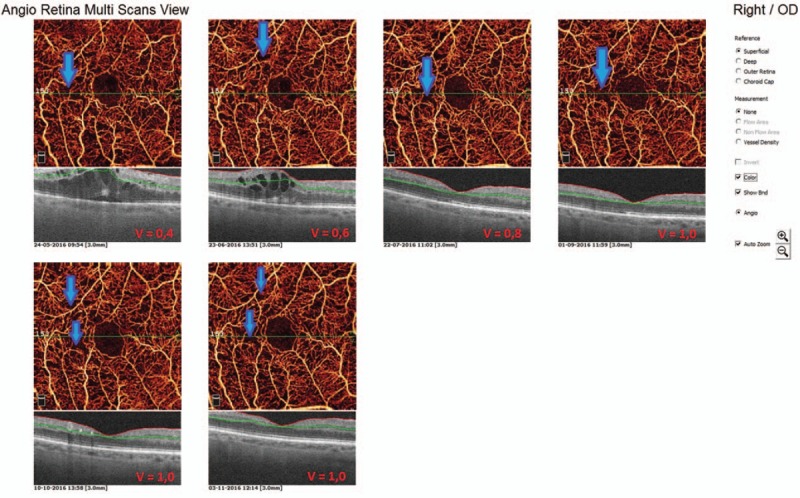
OCTA multiscan view of superficial capillary plexus in a 70-year-old patient with type 2 diabetes mellitus and NPDR. The capillary nonperfusion areas outside FAZ are well visible in all the pictures. The vessels around FAZ are rarefied. After 1st anti-VEGF injection a substantial decrease in retinal edema is to observe.

**Figure 5:**
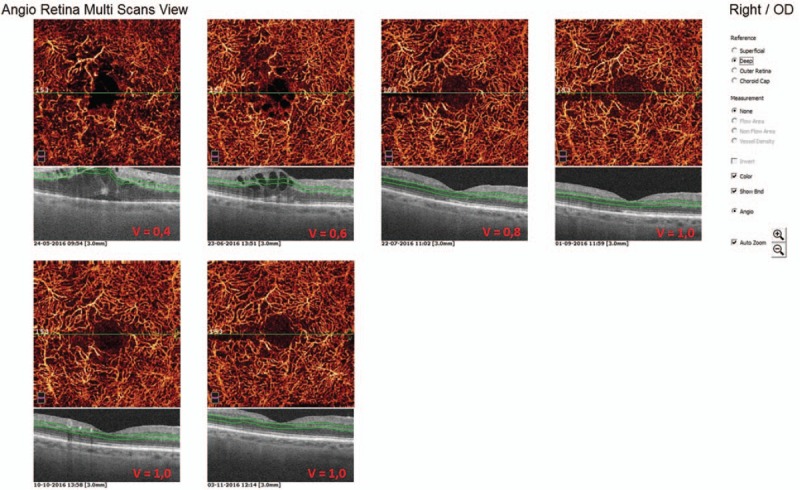
Multiscan view of deep capillary plexus at the baseline and after each intravitreal anti-VEGF injection in a 70-year-old man with type 2 diabetes. Numerous alterations are present, such as irregular vessel pattern due to presence of edema cysts, vessel dilatation, sparse capillaries. After the series of anti-VEGF we observed some improvement of the OCTA capillary picture in DCP comparing to the baseline picture.

**Figure 6:**
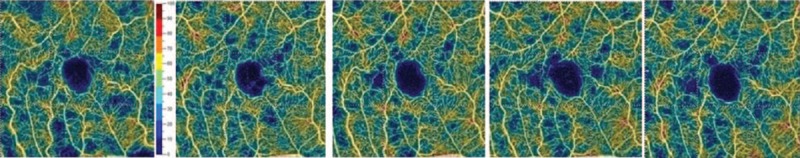
The picture is showing vascular density color perfusion maps generated for superficial capillary layer. The vessel density mapping in second presented case of patient with diabetes type II and DR has shown no difference at the baseline and after intravitreal injections. The mean value of perfused vessels was 45.32%.

The second case is a 70-year-old man with type 2 DM treated with insulin for 10 years. He was diagnosed with DR and DME and received 5 anti-VEGF intravitreal injections in his right eye. This patient presented a very good response to the treatment. Already after first anti-VEGF intravitreal injection the substantial decrease in CRT was to observe. In structural OCT B-scans we observed the normalization of fovea's morphology, the intraretinal edema disappeared. This image findings correlated with visual outcomes: BCVA improved from 0.6 at the baseline, to 0.8 after 2nd and to 1.0 after 3rd intravitreal injection. In angioflow multiscan view of SCP a capillary nonflow areas outside FAZ are well visible in all the pictures (Fig. 4). In scans of DCP more alterations are to observe, such as irregular pattern of vessel walls, vessel dilatation, numerous cysts of retinal edema (Fig. 5). As the CRT decreases after 1st intravitreal injection the cystic changes in DCP scans are less numerous and the vessel pattern normalizes compared to the baseline picture, but the capillaries remain sparse and irregular. The vascular density color perfusion maps for a whole macular image before each injection are showing no difference in vessel density perfusion. The mean value was 45.32% (Fig. 6). Table 2 shows how the CRT values were changing after each intravitreal anti-VEGF injection. The CRT has dropped significantly from 553 to 261 μm. As the values in table shows, the patient had the optimal range of HbA1c (5.8–6.5%) achieving well control over disease.

**Table 2:**
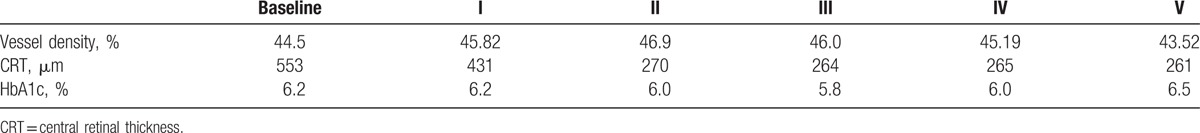
The summary of vessel density, CRT, and HbA1c values at baseline, after 1st, 2nd, 3rd, 4th, and 5th injection in second presented case.

**Figure 7:**
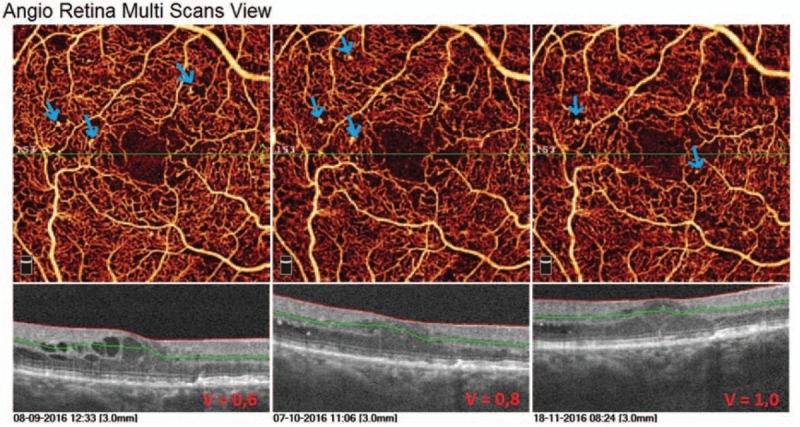
Multiscan view of OCTA of SCP in 70-year-old man with type 2 diabetes mellitus and DR. The arrows are showing well visible microaneurysms, that are mostly present at the edge of capillary nonperfusion areas.

**Figure 8:**
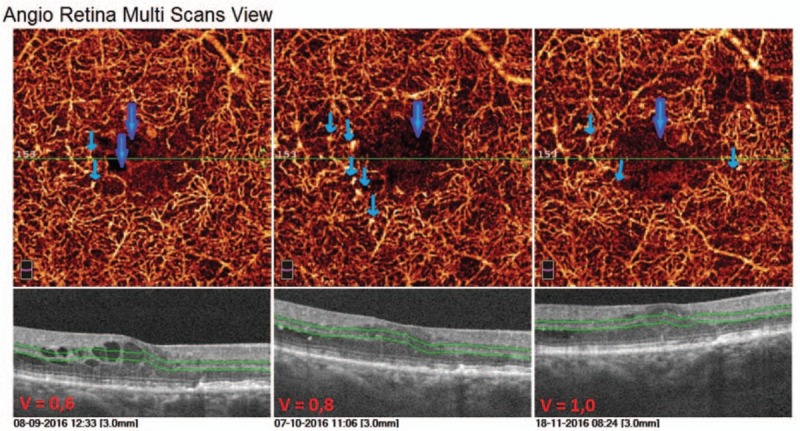
Multiscan view of the DCP in 70-year-old man with type 2 diabetes mellitus and DR. Thick arrows are showing cysts of intraterinal edema. Thin arrows are showing numerous microaneurysms visible as focally dilated, round or fusiform saccular capillaries present mostly next to capillary nonperfusion areas. The architecture of capillary network remains altered. OCT scans are showing slight decrease in macular edema.

**Figure 9:**
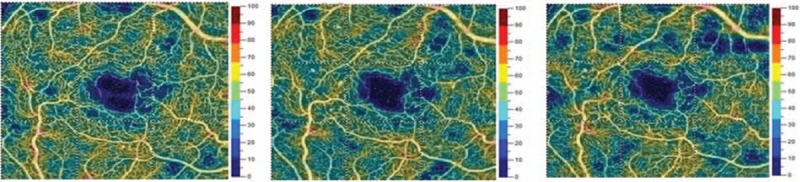
The vessel density mapping in 70-year-old man with diabetes type II and DR has shown no difference at the baseline and after intravitreal injections. The mean value was 46.87%.

Third case is a 70-year-old man with type 2 diabetes and NPDR treated with insulin for 15 years. The patient received a treatment with 3 anti-VEGF intavitreal injections in his right eye. In SCP capillary nonperfusion areas are well visible. At the edge of the nonperfusion areas microaneurysms, which can be identified as focally dilated round, saccular, or fusiform capillaries, are also present (Fig. 7). Microaneurysms were significantly more numerous in the DCP than in the SCP (Fig. 8). In this case in both superficial and DCP the morphology of microvasculature has not change after the treatment. The slight decrease of CRT from 357 to 325 μm is to observe. The clinical outcome in visual acuity was satisfying, BCVA improved from 0.6 to 1.0. Patient achieved a goal of well-controlled diabetes by having a stable HbA1c level (7.0%) before each injection. Although the morphology of retinal microvasculature remained altered, functional improvement was achieved in better BCVA. This phenomenon may be explained by the fact, that anti-VEGF is working more on vessels permeability preventing from leakage, reducing the edema, resulting clinically in better visual acuity. It does not change the density or the morphology of existing vessels, but it can prevent from neovascularization and progression to PDR. The vessel density mapping has shown, similarly to other presented cases, no difference at the baseline and after injections. The mean value was 46.87% (Fig. 9). The change in vessel density, CRT, and HbA1c values is shown in Table 3. HbA1c was stable and equaled to 7%.

**Table 3:**
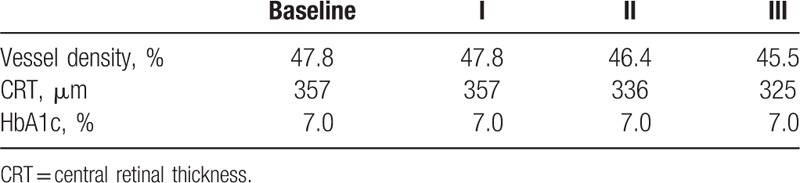
The summary of vessel density, CRT, and HbA1c values at baseline, after 1st, 2nd, and 3rd injection in third presented case.

## 4 Conclusions

In this series of DR cases, OCTA showed specific alterations of the microvascular structure in the superficial and DCP. The superficial and deep vascular plexus were both abnormal in all eyes. In the superficial plexus, a rarefaction of capillaries with nonperfusion areas outside the FAZ were present, whereas in DCP evident microvascular alterations around the small cystoid edema cells were visible in all cases. In the deep vascular plexus, capillary nonperfusion areas outside the FAZ were less seen comparing to superficial capillary network. However, the normal architecture of the DCP was altered in all of the examined eyes, so that the regular pattern of the capillary vortexes could not be identified.^[16,17]^

As previously described by Ishibazawa et al,^[21]^ OCTA allowed visualizing microaneurysms in both superficial and DCP in 3 mm × 3 mm scans. Microaneurysms were identified as focally dilated round, saccular, or fusiform capillaries.^[21]^ Histologic and OCT studies have shown that microaneurysms are mainly located in the inner nuclear layer and its inner/outer edges.^[26,27,28]^ In our cases the microaneurysms were also more numerous in DCP which can suggest that they mainly origin from this plexus.

In all presented cases with DME no capillaries were detected in the cysts in the superficial or DCP. This could be explained either by the development of the cysts preferably in nonperfusion areas or by the displacement of capillaries at the periphery of the cysts. It is also possible, that mechanical pressure associated with the intraretinal cystoid spaces prohibits blood flow in adjacent capillaries. Lastly, it is possible that regions in and around intraretinal fluid actually have occluded or otherwise incompetent capillaries (that presumably led to the DME).^[10]^

In all presented cases, the decrease in CRT that correlated clinically with improvement of visual acuity (BCVA) was observed. The good clinical response to the treatment did not necessarily correlate with an improvement of microvascular changes in OCTA images. That could be explained by the fact, that anti-VEGF is working on inflammatory factors and vessel hyperpermeability preventing also from leakage, thus we could see its effect in reduction of retinal edema. With decrease in cystic changes of edema in OCTA scans the capillaries in SCP and DCP were more visible. Many proinflammatory factors play role in pathogenesis of DR. Well-controlled glycemia is also very significant for the therapeutic success. Our presented cases had well-controlled diabetes as HbA1c values show. Certainly, the morphology of the retina at the baseline and length of retinal edema duration are also very significant for the later clinical outcome after the treatment.

Vascular perfusion maps demonstrate the perfusion density changes as the DR progresses (from mild NPDR, through severe NPDR to PDR). In presented cases the perfusion density level for the whole macular area in 3 mm × 3 mm scans shown in density maps (obtained automatically by the angioanalytics software) remained stable between injections (there were no differences between injections). The longer observation time of the vessel density perfusion maps and capillary nonperfusion areas would allow the evaluation of the DR progression in these patients. Future advances of OCTA software may allow automatic measurement of these areas and automated capillary density mapping for the diagnosis and monitoring of DR.^[19]^

Although the usefulness of OCTA to monitor the treatment of DR is still debated, there are publications showing, that the assessment of capillary nonperfusion areas on OCTA could be a clinically relevant parameter for DR progression monitoring.^[29]^ The better resolution of OCTA then FA and the ability to visualize the DCP are giving us more information about retinal microvasculature and could help in better understanding the pathogenesis of DR.

Summing up, OCTA as dyeless, quick, and noninvasive method of detecting microvascular and ischemic changes in DR might be a useful tool in observing progression of the disease and response to treatment in daily clinical practice.
